# The TRAF6-Akt axis is required for efficient lytic replication of KSHV

**DOI:** 10.1371/journal.ppat.1014557

**Published:** 2026-09-01

**Authors:** Zhenshan Liu, Qingyu Guo, Weili Wang, Hongjia Lu, Tianyu Yu, Tingting Li, Qiming Liang

**Affiliations:** 1 Institute of Pediatric Infection, Immunity, and Critical Care Medicine, Shanghai Children’s Hospital, Shanghai Jiao Tong University School of Medicine, Shanghai, China; 2 Shanghai Institute of Immunology, Department of Immunology and Microbiology, Key Laboratory of Cell Differentiation and Apoptosis of Chinese Ministry of Education, Shanghai Jiao Tong University School of Medicine, Shanghai, China; 3 Department of Thyroid Surgery, China-Japan Union Hospital of Jilin University, Changchun, Jilin, China; 4 State Key Laboratory of Developmental Biology of Freshwater Fish, Human International Joint Laboratory of Animal Intestinal Ecology and Health, Laboratory of Animal Nutrition and Human Health, Hunan Provincial Key Laboratory of Animal Intestinal Function and Regulation, College of Life Sciences, Hunan Normal University, Changsha, Hunan, China; 5 Shanghai Key Laboratory of Severe Respiratory Infection, Shanghai, China; Brigham and Women’s Hospital, UNITED STATES OF AMERICA

## Abstract

TRAF6 is traditionally recognized as an antiviral ubiquitin E3 ligase that positively regulates the production of type I interferon and inflammatory cytokines. However, our study reveals that TRAF6 also plays a crucial role in the lytic replication of Kaposi’s sarcoma-associated herpesvirus (KSHV). Mechanistically, during KSHV lytic replication, TRAF6 mediates the K63-linked polyubiquitination and activation of Akt, which is required for the efficient viral replication. Disruption of TRAF6 or Akt expression through CRISPR-mediated knockout, or inhibition of TRAF6 or Akt with small molecule inhibitors, reduces KSHV replication efficiency. Conversely, expression of constitutively active Akt can rescue the impaired replication caused by TRAF6 deficiency. Notably, the TRAF6-Akt axis is also required for the lytic replication of Epstein-Barr virus but not for Human cytomegalovirus. These findings highlight the role of the TRAF6-Akt axis in the life cycle of oncogenic herpesviruses and suggest potential therapeutic targets for related diseases.

## Author summary

Kaposi’s sarcoma-associated herpesvirus (KSHV) and Epstein-Barr virus (EBV) are cancer-causing viruses that establish lifelong infections in humans. To survive and multiply, these viruses must cleverly manipulate the host cell they infect. A host protein called TRAF6 is traditionally known as an immune system “defender” that helps the body fight off viral infections by triggering inflammation. However, our study reveals a surprising twist: these cancer-causing herpesviruses actually hijack TRAF6 to promote their own replication. We found that during the lytic phase of KSHV infection, TRAF6 acts as a switch to turn on another host protein called Akt. This TRAF6-Akt axis provides an essential signal that allows the virus to multiply efficiently.

## Introduction

The World Health Organization estimates that 15–20% of human cancers are attributable to oncogenic virus infections, with Kaposi’s sarcoma-associated herpesvirus (KSHV) and Epstein-Barr virus (EBV) identified as oncogenic herpesviruses [1,2]. KSHV is the causative agent of Kaposi’s sarcoma, primary effusion lymphoma, and multicentric Castleman’s disease [3,4], while EBV is linked to a broader range of cancers, including Burkitt lymphoma, Hodgkin lymphoma, gastric cancer, and nasopharyngeal carcinoma [5]. Similar to other herpesviruses, both KSHV and EBV establish latency and undergo lytic replication during their lifecycle [6]. While latent infections are closely associated with tumor development, lytic replication is essential for the production of infectious progeny [3,7]. Both viruses encode over 80 open reading frames (ORFs) and numerous viral microRNAs that manipulate cellular processes to create an environment conductive to viral replication and oncogenesis [8,9]. In addition to virus-encoded proteins, host cellular factors also play critical roles in viral lytic replication and pathogenesis.

Tumor necrosis factor receptor (TNFR)-associated factor 6 (TRAF6) is RING finger domain-dependent ubiquitin E3 ligase that plays a critical role as an adaptor in mediating various protein-protein interactions via its TRAF domain [10]. Unlike other members of the TRAF family, TRAF6 preferentially binds to consensus X-X-P-X-E-X-X-aromatic/acidic motifs in its interacting partners via its C-terminal TRAF domain, which is essential for activating downstream signaling pathways [11]. TRAF6 is broadly expressed across mammalian tissue and is highly conserved across species. Its ubiquitin E3 ligase activity catalyzes K63-linked poly-ubiquitination on diverse substrates, which is crucial for intracellular signal transduction in pathways such as TLR/IL-1R, RLR, TNFR, TGFβRI, TCR, and IL-17R [10]. Additionally, TRAF6 promotes the K63-linked poly-ubiquitination of Akt, facilitating its membrane localization and activation in response to growth factors [12]. Beyond attaching poly-ubiquitin chains to target proteins, TRAF6 also catalyzes the formation of unanchored K63-linked poly-ubiquitin chains, which interacts with TAB2/TAB3 and are necessary for TAK1 activation [13]. Autoubiquitination on Lysine 124 (K124) is often used as an indicator of TRAF6 activation, and the deubiquitinating enzymes such as A20 and CYLD have been shown to negatively regulate TRAF6-mediated ubiquitination [14–16].

TRAF6 plays a vital role in antiviral responses by activating signaling pathways that lead to the production of type I interferons (IFN) and the expression of inflammatory cytokines [17]. As a scaffolding protein, TRAF6 recruits downstream kinases such as TBK1 and IKK family members (IKKα, IKKβ, and NEMO), which are essential for activating transcriptional factors like IRF3 and NK-κB [18]. TRAF6 catalyzes K63-linked poly-ubiquitination on the three C-terminal lysine residues of IRF7, the key regulator of type I IFN production, which is necessary for IRF7 activation [19]. Conversely, various viruses have evolved strategies to modulate TRAF6 activity to facilitate their replication and pathogenesis. For example, Human Parainfluenza virus type 2 V protein inhibits TRAF6-mediated ubiquitination of IRF7, thereby preventing type I IFN production [20]. Zika virus NS1 protein interacts with TRAF6 and induces its autophagic degradation [21]. Epstein-Barr virus LMP1 protein directly forms a complex with TRAF6, leading to the activation of NF-κB and JNK, which promote B cell transformation and oncogenesis [22]. Additionally, HIV-1 Tat protein interacts with TRAF6 to activate NF-κB, enhancing HIV-1 transcription [23]. However, the role of TRAF6 in the replication and pathogenesis of oncogenic herpesviruses remains unclear.

The PI3K/Akt signaling pathway is a central hub frequently hijacked by KSHV. Previous studies have demonstrated that KSHV-encoded proteins, including K1, K15, and vGPCR, constitutively activate Akt to promote the survival of infected cells [24–26]. Similarly, TRAF6 has been identified as a critical adaptor in KSHV-mediated signaling, particularly in the context of vFLIP-induced NF-κB activation during latency [27]. However, the specific orchestration of a TRAF6-Akt signaling axis during the transition to lytic replication and its subsequent impact on viral biosynthesis remain less understood.

In this study, we identified TRAF6 as an essential host factor for the lytic replication of oncogenic herpesviruses. TRAF6 is necessary for Akt activation during KSHV lytic replication, as inhibition of Akt activation with specific inhibitors or genetic knockout of Akt markedly reduced viral replication. Furthermore, overexpression of constitutively active Akt restored KSHV lytic replication in TRAF6-deficient cells, indicating that the TRAF6-Akt axis positively regulates KSHV lytic cycle progression. Importantly, we found that inhibition of TRAF6 or Akt also reduces the replication efficiency of EBV, but not Human cytomegalovirus (HCMV), suggesting the critical role of TRAF6-Akt axis in oncogenic herpesviruses.

## Results

### TRAF6 deficiency inhibits KSHV lytic replication

**Fig 1:**
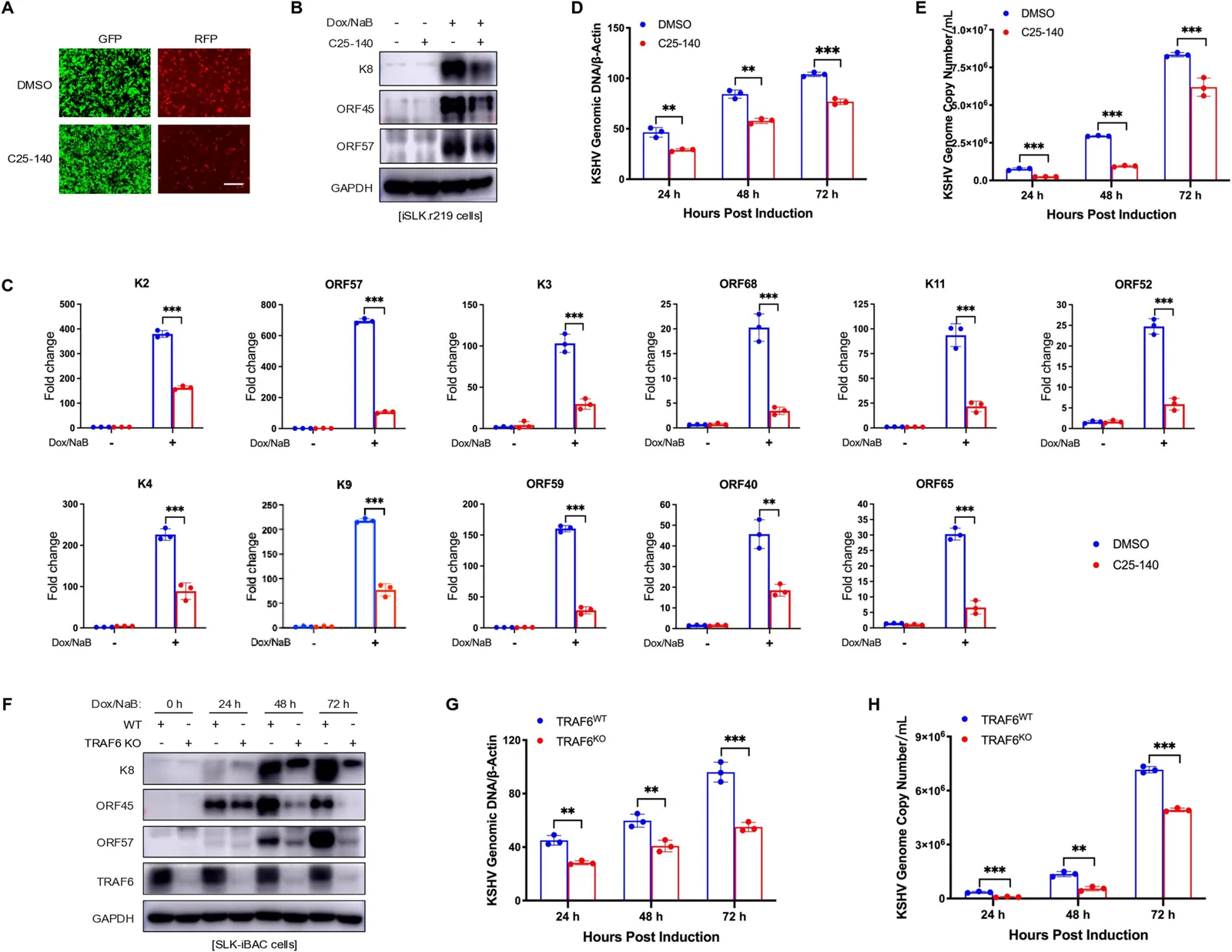
Abrogation of TRAF6 inhibits KSHV lytic replication. **(A–E)** The TRAF6 inhibitor C25-140 limited KSHV lytic replication. The iSLK.r219 cells were treated with doxycycline (Dox, 2 μg/ml) and sodium butyrate (NaB, 1 mM) to induce lytic replication in the presence of C25-140 (30 μM) or DMSO control for 48 h. The GFP and RFP images were captured by Keyence microscope (BZ-X800) (A). Scale bar, 100 μm. Cell lysates were collected and the viral protein expression were determined by immunoblotting (IB) with indicated antibodies (B). Indicated viral gene expressions at mRNA level were determined by RT-qPCR (C). Total DNA was isolated from the cell lysates (D) or the culture supernatant (E) of indicated cells and viral genomic DNA was quantified by qPCR. **(F-H)** TRAF6 knockout blocks KSHV lytic replication in SLK-iBAC cells. SLK-iBAC and SLK^TRAF^ [6] ^KO^-iBAC cells were treated with Dox/NaB for 48 h to induce KSHV lytic replication. Cell lysates were collected and subjected to IB with indicated antibodies (F). Total DNA was isolated from the cell lysates (G) or the culture supernatant (H) of indicated cells and viral genomic DNA was quantified by qPCR. Data represent the means of three independent experiments; Mean ± SD; **p < 0.01, and ***p < 0.001 by one-way ANOVA in (C-E, G-H).

From our previous small-molecule inhibitor screening using iSLK.r219 cell model upon doxycycline-induced lytic replication, we found that C25-140, an inhibitor that disrupts TRAF6-Ubc13 interaction, significantly reduced KSHV lytic replication in iSLK.r219 cells (Fig 1A). iSLK.r219 cells carries latent KSHV and constitutively express GFP, whereas lytic reactivation drives the RFP expression since it is under the control of a lytic promoter [28]. The TRAF6 inhibitor C25-140 significantly reduced RFP expression level and lytic gene expressions upon KSHV lytic reactivation in iSLK.r219 cells (Fig 1A, 1B). To further confirm the effects of C25-140 on KSHV lytic replication, we assessed the lytic gene expressions, viral DNA replication, and progeny virus production in iSLK-BAC16 cells upon doxycycline and sodium butyrate treatment. The results showed that C25-140 treatment redued lytic gene expression in mRNA levels, decreased viral genome copies, and inhibited the production of progeny viruses compared to DMSO treatment control (Fig 1C–1E).

To further evaluate the role of TRAF6 in KSHV lytic replication, we knocked out TRAF6 in SLK-iBAC cells by CRISPR-Cas9 [29]. Consistent with TRAF6 inhibitor treatment, TRAF6 knockout also reduced KSHV lytic gene, viral genome copied, and progeny virus production in SLK-iBAC cells upon doxycycline and sodium butyrate treatment (Fig 1F–1H), confirming that TRAF6 is an important host factor for KSHV lytic replication.

### The ubiquitin E3 ligase activity is required for TRAF6-mediated KSHV lytic replication

**Fig 2:**
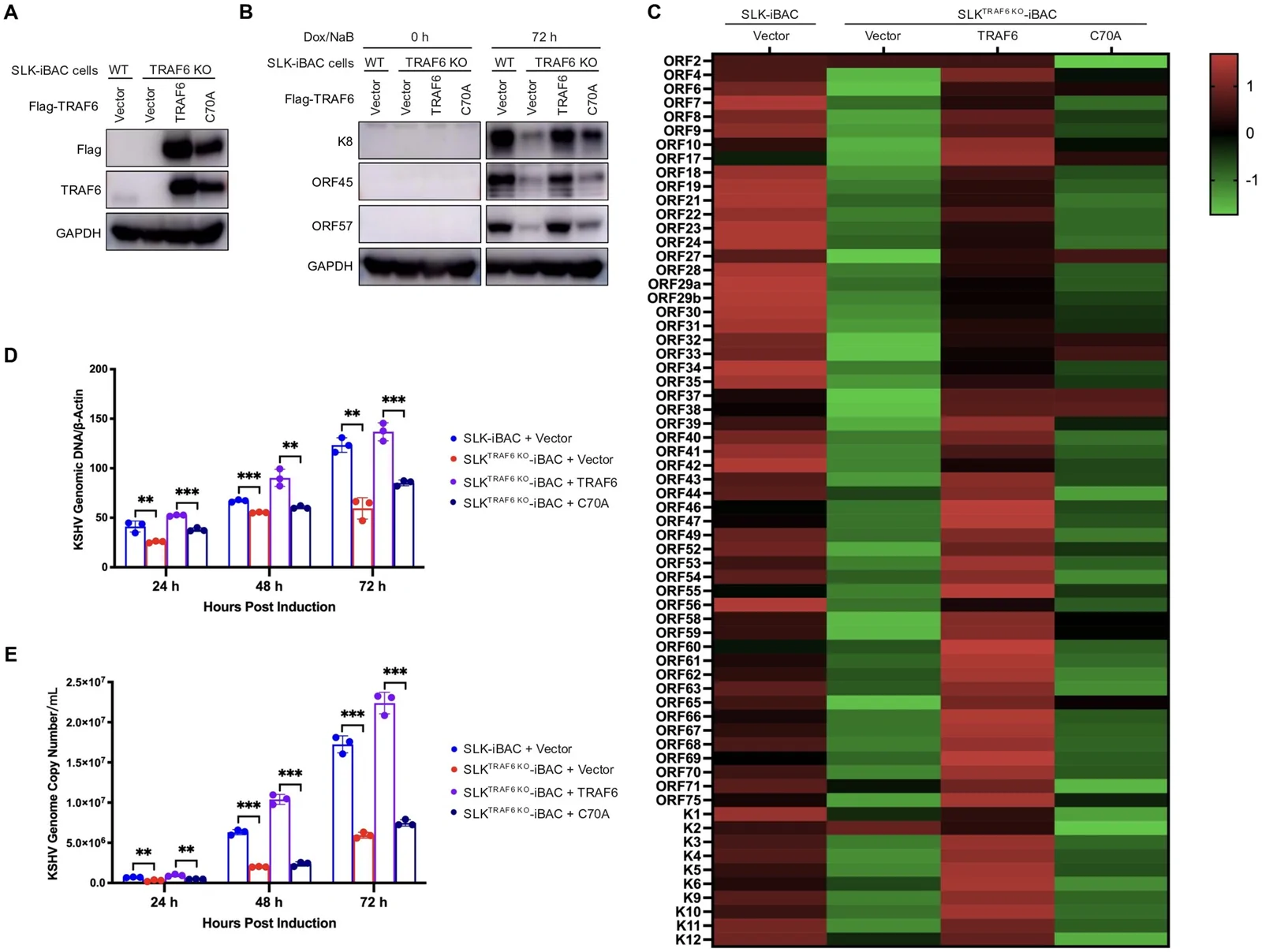
The ubiquitin E3 ligase activity is required for efficient KSHV lytic replication. **(A)** Generation of SLK^TRAF6 KO^-iBAC, SLK^TRAF6 KO^-iBAC-TRAF6^WT^, or SLK^TRAF6 KO^-iBAC-TRAF6^C70A^ cells by CRISPR-Cas9 and lentivirus-mediated stable expression. **(B-C)** The ubiquitin E3 ligase activity of TRAF6 is crucial for KSHV lytic replication. Indicated cell lines were treated with Dox/NaB for 72 h to induce KSHV lytic replication. Cell lysates were collected and subjected to IB with indicated antibodies (B). Indicated SLK-iBAC cell lines were induced with Dox/NaB for 48 h. Total RNA was extracted, reverse-transcribed into cDNA, and used for KSHV whole-genome qPCR array analysis. The ΔCT values for each primer set were calculated and converted to a heatmap using R (C). **(D-E)** The ubiquitin E3 ligase activity of TRAF6 is required for efficient viral genomic DNA replication (D) as well as progeny virus production (E) during KSHV lytic replication. The cell lysates or culture medium containing progeny viruses were collected at indicated time point. Total DNA was isolated and viral genomic DNA was quantified by qPCR. Data represent the means of three independent experiments; Mean ± SD; **p < 0.01, and ***p < 0.001 by one-way ANOVA in (D-E).

TRAF6 functions as a ubiquitin E3 ligase, and cysteine to alanine mutation (C70A) in its RING domain completely abolishes its E3 ligase activity [15]. To investigate the role of its E3 ligase activity in KSHV lytic replication, we reintroduced wild-type TRAF6 (TRAF6^WT^), TRAF6^C70A^, or an empty vector control into SLK^TRAF6 KO^-iBAC cells via lentiviruses transduction (Fig 2A). We then assessed KSHV lytic induction following doxycycline and sodium butyrate treatment. Immunoblot analysis revealed that, at 48 h post-induction, the protein expression levels of lytic genes, such as K8, ORF45, and ORF57 were significantly restored by TRAF6^WT^ in SLK^TRAF6 KO^-iBAC cells, reaching levels compared to those in wild-type SLK-iBAC cells (Fig 2B). In contrast, the TRAF6^C70A^ mutant, which lacks ubiquitin E3 ligase activity, behaved like the vector control and failed to support the expressions of these viral proteins (Fig 2B), indicating that the ubiquitin E3 ligase function of TRAF6 is crucial for KSHV lytic replication. We further systematically compared viral gene expression profiles between TRAF6^WT^ and TRAF6^C70A^ expressing cells by evaluating viral mRNA levels by genome-wide quantitative RT-PCR array at 48 hours post-lytic replication. Consistent with protein expression level of viral genes, TRAF6^WT^ markedly increased the overall KSHV gene mRNA levels in SLK^TRAF6 KO^-iBAC cells, while TRAF6^C70A^ did not (Fig 2C). Additionally, viral DNA copies were higher in SLK^TRAF6 KO^-iBAC cells expressing TRAF6^WT^ compared to those with TRAF6^C70A^ (Fig 2D). Finally, we collected the culture supernatant during KSHV lytic replication to quantify progeny virus production. The absence of TRAF6 reduced viral progeny, but expression of TRAF6^WT^, and not TRAF6^C70A^, restored progeny virus levels (Fig 2E). Collectively, these findings demonstrate that the ubiquitin E3 ligase activity of TRAF6 is required for efficient KSHV lytic replication and progeny virus production.

### TRAF6 is required for Akt activation during KSHV lytic replication

**Fig 3:**
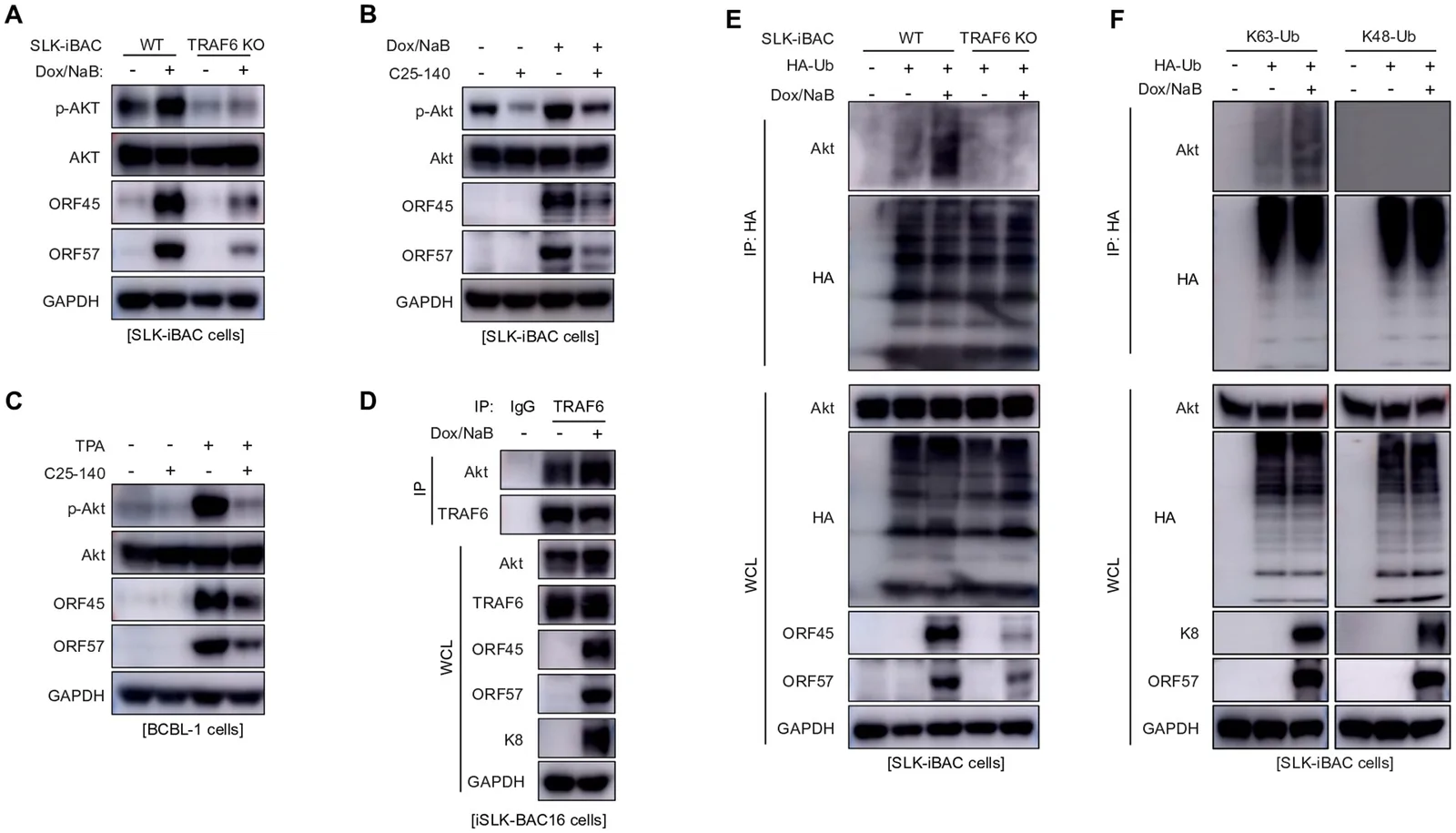
TRAF6 mediates the K63-linked ubiquitination and activation of Akt upon KSHV lytic reactivation. **(A-C)** TRAF6 is necessary for Akt activation during KSHV lytic replication. SLK-iBAC cells and SLK^TRAF6 KO^-iBAC cells were treated with Dox/NaB for 48 h to induce KSHV lytic replication. Cell lysates were collected and subjected to IB with indicated antibodies (A). C25-140 (30 μM) treatment blocks Akt activation upon KSHV lytic reactivation in SLK-iBAC (B) and BCBL-1 cells (C). **(D)** Endogenous interaction between TRAF6 and Akt during KSHV lytic replication in iSLK-BAC16 cells. **(E)** TRAF6 is essential for Akt ubiquitination during KSHV lytic replication. SLK-iBAC and SLK^TRAF6 KO^-iBAC cells stably expressing an empty vector or HA-ubiquitin were induced with Dox/NaB for lytic replication for 48 h, and the cell lysis were collected for IP under denaturing condition and IB with indicated antibodies. **(F)** Akt undergoes K63-linked polyubiquitination during KSHV lytic replication. SLK-iBAC cells stably expressing an empty vector, or HA-ubiquitin (K63 only), or HA-ubiquitin (K48 only) were induced with Dox/NaB for lytic replication for 48 h, and the cell lysis were collected for IP under denaturing condition and IB with indicated antibodies.

TRAF6 mediates K63-linked ubiquitination of Akt, a modification essential for its recruitment to the membrane and subsequent activation [12]. To investigate whether TRAF6 influences Akt activation during KSHV lytic replication, we induced lytic replication in both wild-type SLK-iBAC and SLK^TRAF6 KO^-iBAC cells using doxycycline and sodium butyrate. While the total level of Akt remained unchanged regardless of KSHV reactivation or TRAF6 deficiency, KSHV lytic replication stimulated Akt activation, evidenced as the increased phosphorylation level of Akt compared latent infected cells (Fig 3A). Importantly, TRAF6 knockout significantly abolished Akt phosphorylation upon KSHV lytic replication (Fig 3A). Consistently, treatment with the TRAF6 inhibitor C25-140 significantly reduced Akt phosphorylation during KSHV lytic replication in both SLK-iBAC and BCBL-1 cells (Fig 3B, 3C), indicating that TRAF6 is necessary for Akt activation during KSHV lytic replication.

Furthermore, we assessed whether TRAF6 is required for Akt ubiquitination during KSHV lytic replication. Endogenous interaction between TRAF6 and Akt was readily detected during KSHV lytic replication (Fig 3D). To conveniently assess the Akt ubiquitination, we overexpressed HA-tagged ubiquitin in both wild-type SLK-iBAC and SLK^TRAF6 KO^-iBAC cells, followed by doxycycline-mediated lytic induction. Consistent with Akt phosphorylation, KSHV lytic replication triggered Akt polyubiquitination in wild-type SLK-iBAC cells, whereas TRAF6 deficiency totally abolished Akt ubiquitination in SLK^TRAF6 KO^-iBAC cells (Fig 3E), suggesting that TRAF6 is essential for Akt ubiquitination during KSHV lytic replication. Additionally, the TRAF6-mediated polyubiquitination of Akt was K63-linked but not K48-linked during KSHV lytic replication (Fig 3F). These findings demonstrate that TRAF6 is crucial for Akt ubiquitination and activation in context of KSHV lytic cycle progression.

### Blocking Akt activation abolishes KSHV lytic replication

**Fig 4:**
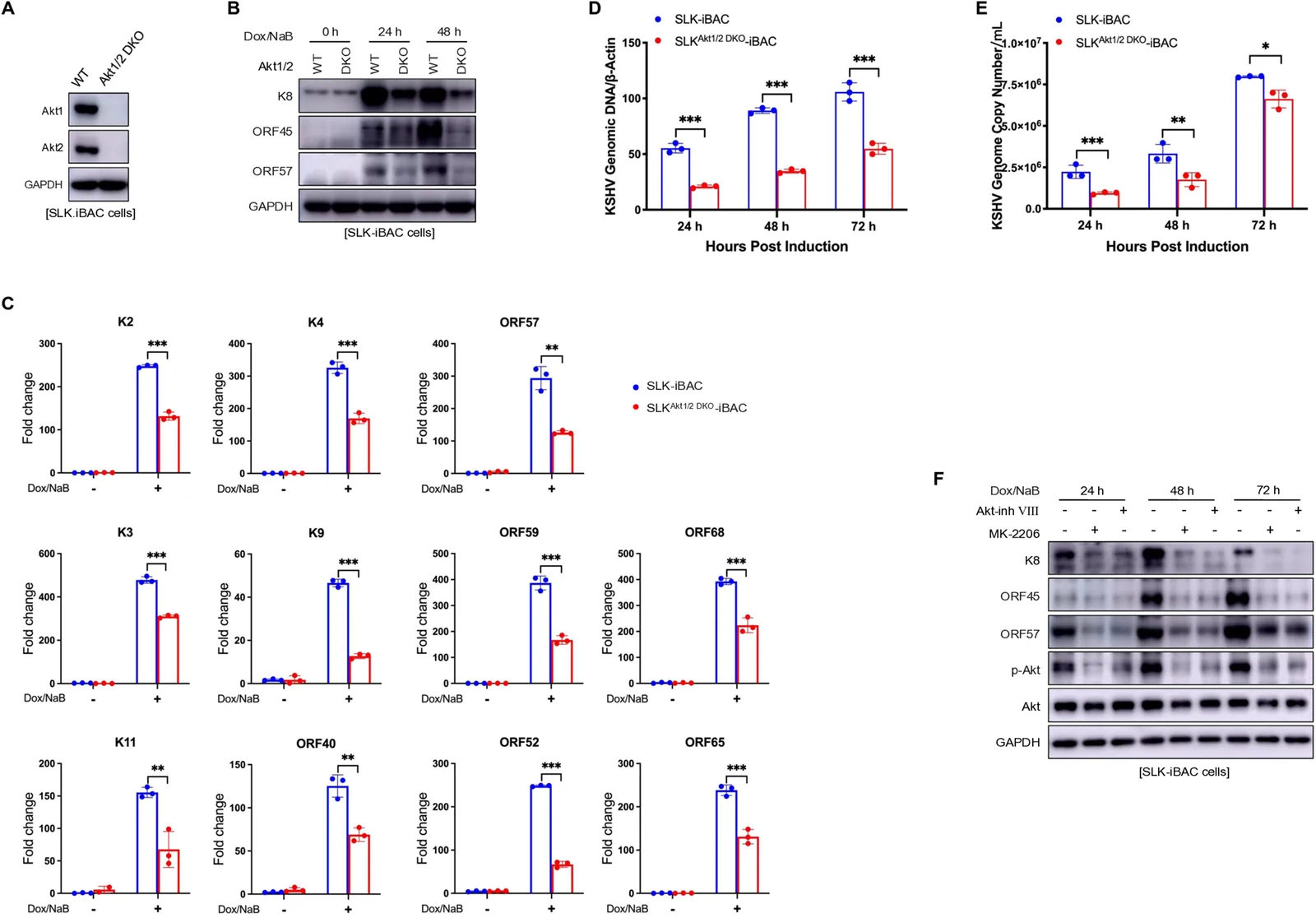
Inhibition of Akt impairs KSHV lytic replication. **(A)** Generation of Akt1/2 double knockout (DKO) in SLK-iBAC cells by CRISPR-Cas9. **(B-E)** Akt is required for efficient KSHV lytic replication in SLK-iBAC cells. SLK-iBAC or SLK^Akt1/2 DKO^-iBAC cells were treated with Dox/NaB to induce KSHV lytic replication. Cell lysates were collected and subjected to IB with indicated antibodies (B). Indicated viral gene expressions at mRNA level were determined by RT-qPCR (C). Total DNA was isolated from the cell lysates (D) or the culture supernatant (H) of indicated cells and viral genomic DNA was quantified by qPCR. **(F)** Akt activation is necessary for KSHV lytic replication. The SLK-iBAC cells were treated with Dox/NaB to induce lytic reactivation in the presence of DMSO, MK-2206 (1 μM), or Akt inhibitor Ⅷ (Akt-inh Ⅷ, 1 μM) for 48 h and the cell lysates were collected and subjected to IB with indicated antibodies. Data represent the means of three independent experiments; Mean ± SD; *p < 0.05, **p < 0.01, and ***p < 0.001 by one-way ANOVA in (C-E).

Given that TRAF6-mediated ubiquitination is essential for Akt activation during KSHV lytic replication across multiple cell infection models, we next investigated whether Akt itself is necessary for efficient KSHV reactivation. We used CRISPR-Cas9 to knock out both Akt1 and Akt2 in SLK-iBAC cells and assessed KSHV replication in both wild-type and Akt1/2 double knockout (DKO) SLK-iBAC (SLK^Akt1/2 DKO^-iBAC) cells following induction with doxycycline and sodium butyrate (Fig 4A). Unlike the robust reactivation observed in wild-type cells, SLK^Akt1/2 DKO^-iBAC cells displayed impaired reactivation, as evidenced by reduced expression of K8, ORF45, and ORF57 detected with specific antibodies (Fig 4B). Additionally, at 48 hours post-reactivation, quantitative analysis of viral gene expression revealed higher levels of immediate early genes (K2, K4, and ORF57), early genes (K3, K9, ORF59, and ORF68), and late genes (K11, ORF40, ORF52, and ORF65) in wild-type SLK-iBAC cells compared to SLK^Akt1/2 DKO^-iBAC cells (Fig 4C). Consistently, viral genome copies were substantially lower in SLK^Akt1/2 DKO^-iBAC cells (Fig 4D). The progeny virus production was also reduced in SLK^Akt1/2 DKO^-iBAC cells compared to wild-type SLK-iBAC cells (Fig 4E), indicating an important role for Akt in KSHV lytic replication.

Furthermore, we examined whether Akt activation is necessary for KSHV lytic replication. We employed Akt specific inhibitors, Akt inhibitor Ⅷ and MK-2206, to block Akt phosphorylation and activation, and evaluated KSHV reactivation in SLK-iBAC cells. Both inhibitors effectively abolished Akt phosphorylation during KSHV lytic replication (Fig 4F). Compared to DMSO controls, treatment with Akt inhibitor Ⅷ or MK-2206 suppressed KSHV lytic replication, as evidenced by reduced expression levels of viral lytic genes, including K8, ORF45, and ORF57, in SLK-iBAC cells (Fig 4F). These results indicate that TRAF6-mediated Akt activation is required for efficient KSHV lytic replication in multiple infection models.

### Expression of constitutive activated Akt rescues KSHV lytic replication in TRAF6 knockout cells

**Fig 5:**
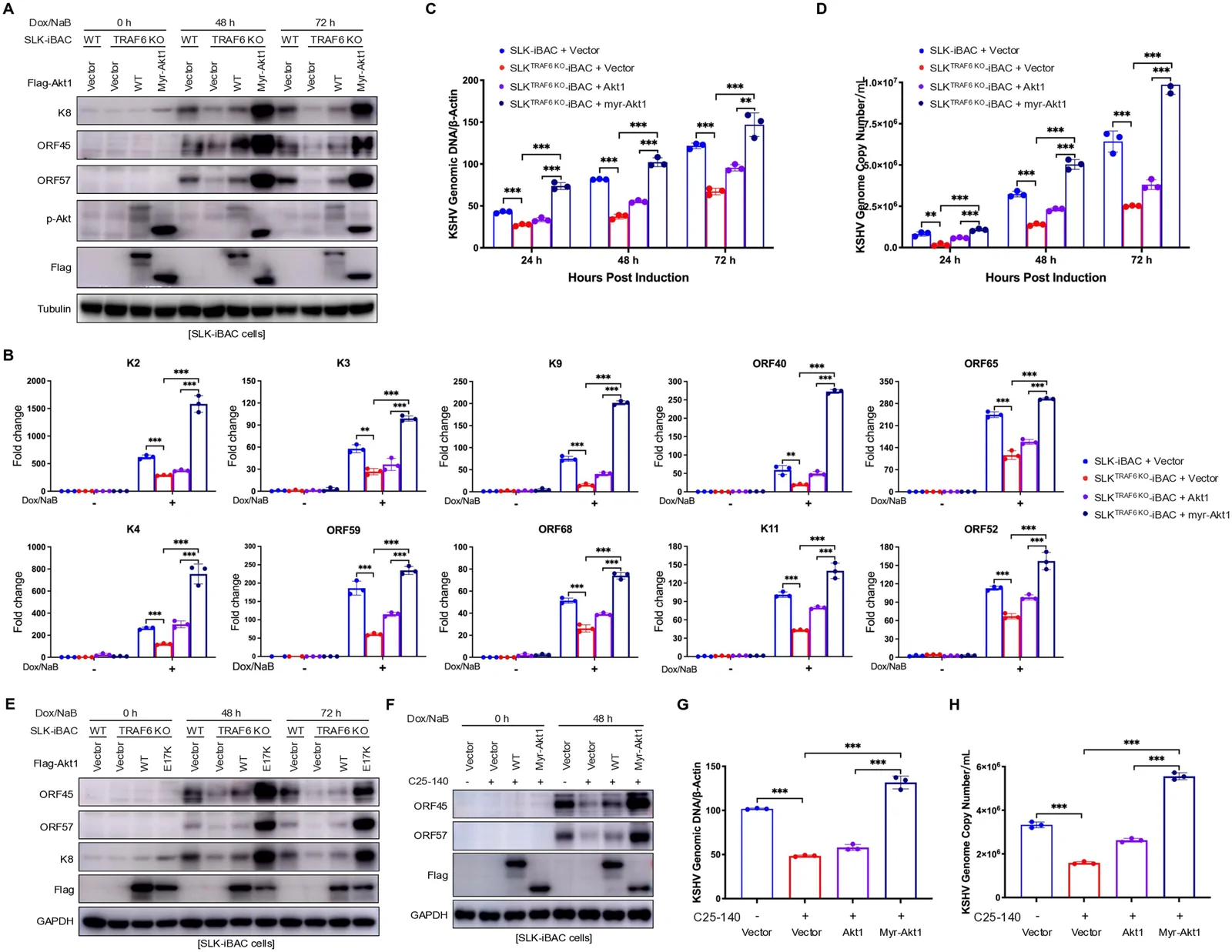
Expression of constitutively active Akt rescue KSHV lytic replication in TRAF6-deficient cells. **(A-D)** Constitutively active Akt (myr-Akt1) restores KSHV lytic replication in TRAF6 deficient cells. SLK-iBAC-Vector, SLK^TRAF6 KO^-iBAC-Vector, SLK^TRAF6 KO^-iBAC-Akt1, SLK^TRAF6 KO^-iBAC-myr-Akt1 stable cell lines were treated with Dox/NaB to induce KSHV lytic replication. Cell lysates were collected and subjected to IB with indicated antibodies (A). Indicated viral gene expressions at mRNA level were determined by RT-qPCR (B). Total DNA was isolated from the cell lysates (C) or the culture supernatant (D) of indicated cells and viral genomic DNA was quantified by qPCR. **(E)** Constitutively active Akt1^E17K^ restores KSHV lytic replication in TRAF6 deficient cells. Similar procedure as in Fig 5A. **(F-H)** Myr-Akt1 restores KSHV lytic replication in C25-140-treated SLK-iBAC cells. SLK-iBAC cells stably expressing an empty vector, Akt1, or myr-Akt1 were induced with Dox/NaB for lytic replication in the presence of C25-140 (30 μM) or DMSO control for 48 h, cell lysates were collected and subjected to IB with indicated antibodies (F). Total DNA was isolated from the cell lysates (G) or the culture supernatant (H) of indicated cells and viral genomic DNA was quantified by qPCR. Data represent the means of three independent experiments; Mean ± SD; **p < 0.01, and ***p < 0.001 by one-way ANOVA in (B-D, G-H).

Since TRAF6-mediated Akt activation is required for efficient KSHV lytic reactivation, we next examined whether constitutive activation of Akt could restore lytic replication in TRAF6 deficient cells. Myristoylation of Akt promotes its association with the membrane, resulting in its constitutive phosphorylation and activation [30]. To generate constitute active Akt, we fused myristoylated sequence (myr) to the N-terminus of Akt1 (myr-Akt1) and assessed its effect of on KSHV lytic replication in SLK^TRAF6 KO^-iBAC cells. In SLK^TRAF6 KO^-iBAC cells, overexpression wild-type Akt1 failed to efficiently restored lytic gene expression, whereas myr-Akt1 significantly increased lytic gene levels, exceeding those observed in wild-type SLK-iBAC cells (Fig 5A, 5B). Additionally, myr-Akt1 rescued viral DNA replication and progeny virus production in SLK^TRAF6 KO^-iBAC cells (Fig 5C, 5D). Similarly, the E17K mutation in Akt1 which confer constitutive activation and is commonly found in various cancers including breast, colorectal, lung, and ovarian cancers [31], showed comparable ability to myr-Akt1 in restoring KSHV lytic replication in SLK^TRAF6 KO^-iBAC cell (Fig 5E). These findings suggest that TRAF6 promotes KSHV lytic reactivation through Akt activation. Next, we evaluated the impact of constitutive active Akt on KSHV lytic replication in SLK-iBAC cells treated with C25-140. Overexpression of myr-Akt1 significantly restored lytic gene expression, viral DNA levels, and progeny virus production in C25-140-treated SLK-iBAC cells, compared to vector controls (Fig 5F, 5G). Collectively, these results demonstrate that the TRAF6-Akt axis is required for efficient KSHV lytic replication.

### The TRAF6-Akt axis is also required for the efficient lytic replication of EBV

**Fig 6:**
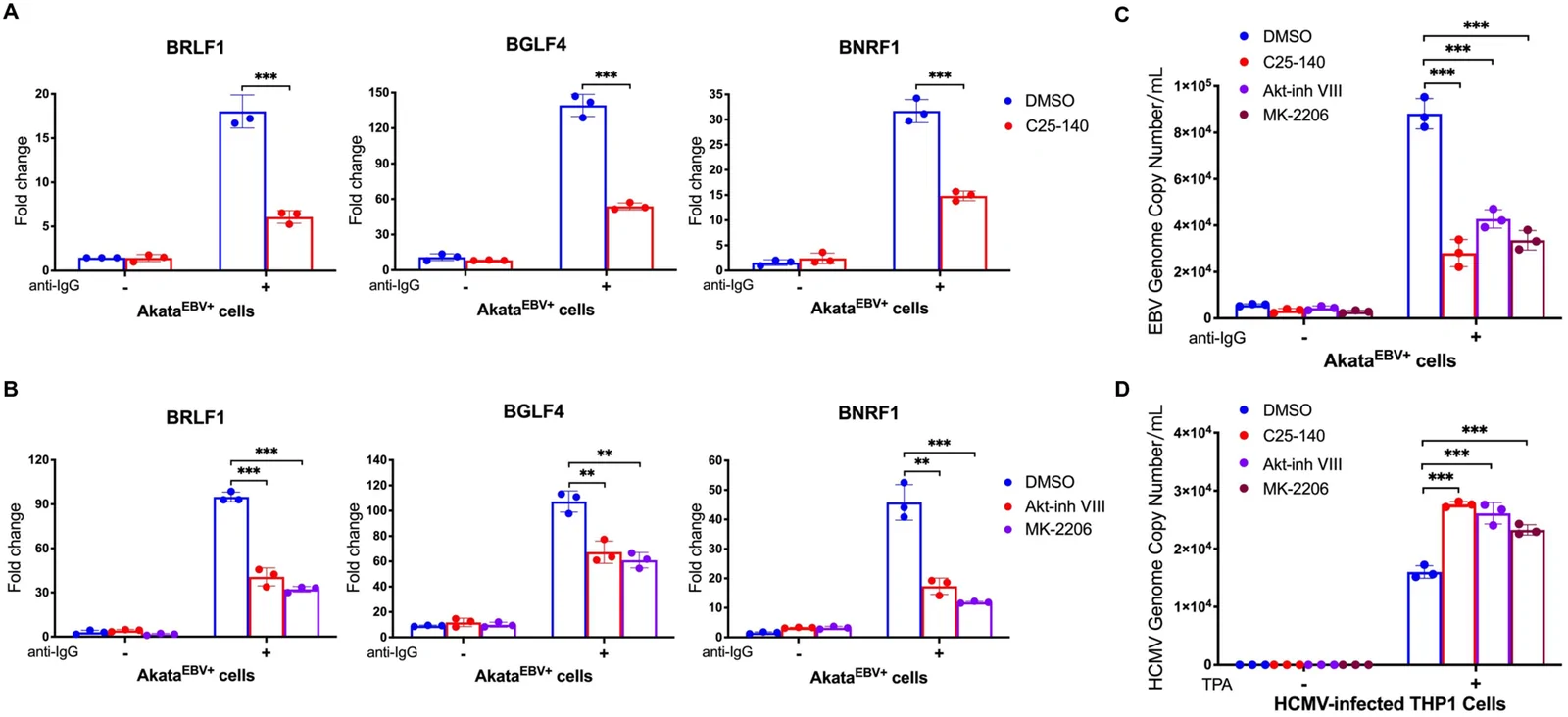
The TRAF6-Akt axis is also required for the lytic replication of EBV. **(A-C)** The TRAF6-Akt axis is required for EBV lytic replication. Akata cells were treated with anti-IgG (10 μg/mL) to induce the lytic replication in the presence of indicated inhibitors for 72 h. Indicated viral gene expressions at mRNA level were determined by qRT-PCR (A-B). The viral genome copy numbers of progeny viruses were quantified by qRT-PCR **(C)**. (D) The TRAF6-Akt axis is not required for the lytic replication of HCMV. HCMV-infected THP-1 cells were treated TPA to induce THP-1 differentiation and HCMV lytic reactivation with the indicated inhibitors or DMSO control. HCMV lytic replication was quantified by the viral genome copied numbers of progeny viruses at 96 h post-induction by qRT-PCR. Mean ± SD; **p < 0.01, and ***p < 0.001 by one-way ANOVA.

Finally, we investigated whether the TRAF6-Akt axis plays a conserved role across other herpesviruses. Given that both EBV and KSHV are members of the γ-herpesvirus subfamily, we first evaluated the impact of TRAF6 inhibition (C25-140) and Akt inhibition (Akt inhibitor Ⅷ or MK-2206) on EBV lytic reactivation using latent infected Akata cells. Following induction with anti-IgG antibody, both TRAF6 and Akt inhibitors suppressed EBV lytic replication. This was evidenced by a significant reduction in both viral lytic gene transcription (Fig 6A, 6B) and the release of progeny virions into the culture supernatant (Fig 6C), recapitulating the inhibitory effects observed in KSHV.

To determine if this requirement extends to the β-herpesvirus subfamily, we utilized the THP-1 cell model to study HCMV lytic reactivation. THP-1 monocytes are conditionally permissive for HCMV and the lytic reactivation is typically coupled with TPA-induced differentiation into macrophages [32,33]. We treated HCMV-infected THP-1 cells with TRAF6 or Akt inhibitors prior to TPA stimulation. In striking contrast to our findings in γ-herpesviruses, neither the TRAF6 nor the Akt inhibitor hampered HCMV lytic reactivation (Fig 6D). Collectively, these findings demonstrate that the TRAF6-Akt axis is a critical, conserved regulator of lytic replication for γ-herpesvirus (KSHV and EBV), but is not required for the β-herpesvirus HCMV.

## Discussion

Ubiquitination is a highly conserved, three-enzyme process involving E1 (activating), E2 (conjugating), and E3 (ligating) enzymes, with the E3 ligase providing substrate specificity [34]. Both KSHV and EBV tightly interact with the host ubiquitin system to promote viral replication and pathogenesis. KSHV encodes its own ubiquitin E3 ligases, such as RTA, K3, and K5, which facilitate immune evasion and lytic reactivation: RTA directly ubiquitinates substrates like IRF7 and MyD88, targeting them for degradation [35,36]; K3 and K5 catalyze the ubiquitination of MHC-1, removing it from the cell surface and thereby preventing cytotoxic T-cell attack [37,38]. Additionally, KSHV exploits host ubiquitin E3 ligases to target cell factors for ubiquitination and degradation. For example, KSHV LANA enhances the activity of the host E3 ligase RLIM, leading to the degradation of RLIM substrates such as LDB1 and LMO2 [39]. KSHV RTA interacts with the host E3 ligase complex RNF20/40 to drive lytic reactivation [40]. Similarly, EBV RTA interacts with host E3 ligase RNF4 to regulate EBV lytic replication [41]. EBV LMP2A binds to the host ubiquitin E3 ligase AIP4, disrupting B-cell signaling [42,43]. In this study, we identified that the cellular ubiquitin E3 ligase TRAF6 as an important host factor for the lytic replication of both KSHV and EBV. Although TRAF6 has many characterized substrates [10], we found that TRAF6-mediated ubiquitination and activation of Akt are crucial for efficient viral lytic replication. Notably, expression of constitutively active Akt can rescue viral replication in TRAF6 deficient cells, highlighting the critical role of the TRAF6-Akt axis in the life cycle of oncogenic herpesviruses.

Although playing critical roles in host innate immune signaling, some cellular antiviral factors, such as MAVS, IKKβ, and IKKε, are actually required for efficient viral replication in γ-herpesvirus. For instance, the KSHV-encoded SUMO E3 ligase ORF45 catalyzes the SUMOylation of IKKε and hijacks it to disrupt the formation of PML nuclear body, thereby facilitating viral lytic replication [44,45]. Murine γ-Herpesvirus 68 also exploits MAVS and IKKβ to inhibit NF-κB activation and suppress antiviral cytokine production [46]. Like MAVS and IKKs, TRAF6 is a key host factor that promotes antiviral innate immunity by activating signaling pathways, particularly the NF-κB pathway, leading to the production of type I IFNs [10]. However, we found that TRAF6 is required for the life cycle of oncogenic herpesviruses such as KSHV and EBV, but not HCMV. This suggests that certain viral factors may manipulate TRAF6 to counteract its antiviral role to instead promote viral replication, effectively converting it into a proviral factor. Further investigation is required for illustrate how KSHV or EBV modulates the TRAF6-Akt axis upon lytic reactivation.

The activation of the PI3K/Akt signaling pathway represents a strategic metabolic hijack by KSHV to ensure the success of the lytic cycle. While Akt is well-known for its role in maintaining latency, its activation during the lytic surge is essential for meeting the massive biosynthetic demands of virion production. KSHV-encoded proteins, such as K1 and K15, contain signaling motifs that constitutively activate the PI3K/Akt axis [24–26]. The lytic switch protein RTA has been shown to synergize with Akt signaling to enhance its own transcriptional activity. Additionally, by integrating these signals, Akt activation creates a cellular environment optimized for rapid nucleotide production and protein synthesis.

In addition to regulating lytic replication, the TRAF6-Akt axis may also play a role in the tumorigenesis of oncogenic herpesviruses. For example, EBV-encoded oncogene LMP1 directly interacts with TRAF6 to activate NF-κB signaling, promoting the development of B cell lymphoma [22,47]. Conversely, EBV BPLF1 can deubiquitinate TRAF6, inhibiting NF-κB pathway activation and thereby facilitating viral lytic DNA replication [48], indicating a complex modulation of TRAF6-mediated signaling during EBV replication and pathogenesis. Both KSHV and EBV infections activate the PI3K/Akt pathway, leading to increased cell proliferation, reduced apoptosis, and enhanced cytoskeleton dynamics [49–52]. EBV proteins LMP1 and LMP2A are known to mediate the activation of the PI3K/Akt pathway, contributing to viral oncogenesis [52]. Since both TRAF6 and Akt are enzymes, they can be targeted by small-molecule inhibitors. Our results demonstrate that these inhibitors suppress the lytic replication of both KSHV and EBV. Currently, dual inhibitors targeting PI3K/Akt and its downstream effector mTOR are undergoing clinical trials [53–56], and they may be incorporated into future treatments for KSHV- or EBV-associated cancers.

## Methods

### Cells

HEK293T, HFF and A549 cells were maintained in Dulbecco’s modified Eagle’s medium (DMEM). iSLK.r219 and iSLK-BAC16 cells were maintained in complete DMEM medium with 400 μg/mL hygromycin, 10 μg/mL puromycin, and 250 μg/mL G418. SLK-iBAC, SLK-iBAC^TRAF6 KO^, and SLK-iBAC^Akt1/2 DKO^ cells were maintained in complete DMEM medium with 400 μg/mL hygromycin. BCBL-1 and Akata cells were cultured in RPMI 1640 medium. All medium was supplemented with 10% fetal bovine serum (FBS), 2 mM L-glutamine, 100 units/ml penicillin and 100 mg/ml streptomycin at 37°C in a 5% CO_2_ incubator. Stable cell lines were generated using a standard selection protocol with puromycin (2 μg/mL) or blasticidin (10 μg/mL). Doxycycline (2 μg/mL) and sodium butyrate (1 mM) treatment was used to induce KSHV lytic replication in SLK-iBAC, iSLK.r219, or iSLK-BAC16 cells. TPA (20 ng/mL) treatment was used to induce KSHV lytic replication in BCBL1 cells. Anti-human IgG (10 μg/mL) treatment was used to induce EBV lytic replication in Akata cells.

### Generation of stable knockout cell lines

sgRNAs were cloned into LentiCRISPR V2-Puro (Addgene #52961) and lentiCRISPR v2-Blast (Addgene, #112233). Lentiviruses were generated by co-transfection of LentiCRISPR V2 plasmid carrying corresponding sgRNAs with three packaging plasmids into HEK293T cells and the culture supernatant was collected at 72 h post-transfection. Lentivirus carrying both Cas9 and sgRNA were utilized to infected SLK-iBAC or BCBL-1 cells and puromycin or blasticidin selection were performed at 48 h post-infection for at least 2 weeks. The knockout efficiency of the polyclonal cell lines was confirmed by immunoblot with specific antibodies. The sequences of the sgRNA are: 5’- AGGCGTATTGTACCCTGGAA -3’ for TRAF6; 5’- GACAACCGCCATCCAGACTG -3’ for Akt1; 5’- CATCGAGAGGACCTTCCACG -3’ for Akt2.

### Inhibitor treatment

iSLK.r219, iSLK-BAC16, SLK-iBAC, or BCBL-1 cells were pre-treated with C25-140 (30 μM), AKT inhibitor Ⅷ (1 μM), or MK-2206 (1 μM), or DMSO control for 6 h in the complete medium and then induced for the lytic replication for 48 h. The viral replication was quantified by the levels of viral transcripts, viral genomic DNA, and progeny virus production.

### Antibodies and chemicals

HRP anti-HA (#901519) and HRP anti-Flag (#637311) antibodies were purchased from BioLegend. Anti-Akt1 (#A17909), Akt2 (#A24009), and GAPDH (#A19056) antibodies were purchased from ABclonal. Anti-TRAF6 (#67591), Akt (#4691), and Phospho-Akt (Ser473) (#4060) antibodies were purchased from Cell Signaling Technology. Anti-KSHV ORF57 (#sc-135746) were purchased from Santa Cruz. Monoclonal antibodies against ORF45 and polyclonal antibodies against K8 were described previously [45,57]. EZview Red Anti-HA M2 Affinity Gel (#E6779), sodium butyrate (#B5887), and TPA (#P1585) were ordered from Sigma. Doxycycline (#S4163) was ordered from Selleck. ClonExpress II One Step Cloning Kit (#C122-01) and HiScript II Q RT SuperMix for qPCR (+gDNA wiper) (#R223-01) were purchased from Vazyme Biotech. Lipofectamine 3000 (#3000015) was purchased from Thermo Fisher Scientific. C25-140 (HY-120934), Akt inhibitor Ⅷ (HY-10355), and MK-2206 (HY-108232) were purchased from MedChemExpress, AffiniPure^TM^ F(ab’)^2^ Fragment Goat Anti-Human IgG+ IgM (H + L) (#109-006-127) was ordered from Jackson ImmunoResearch.

### Plasmid constructs

The cDNAs for TRAF6 and Akt1 were provided by the Core Facility of Basic Medical Sciences, Shanghai Jiao Tong University School of Medicine. The TRAF6^C70A^ and Akt1^E17K^ point mutations were generated using ClonExpress II One Step Cloning Kit (Vazyme Biotech, #C122-01). Myr-Akt1 was generated by fusing Myr sequence (MGSSKSKPKDPSQR) to the N-terminus of Akt1 Δ4-129 [58,59]. For generating stable cell lines, TRAF6, TRAF6^C70A^, myr-Akt, and Akt1^E17K^ were cloned into pCDH vector with Flag tag, and ubiquitin was cloned into pCDH vector with HA tag. The gRNA PAM sequences of TRAF6 and Akt1 were synonymously mutated to avoid the gene silencing mediated by gRNAs. All constructs were sequenced using an ABI PRISM 377 automatic DNA sequencer to verify 100% correspondence with the original sequence.

### Immunoprecipitation and immunoblotting

Immunoprecipitation in denaturing conditions for detecting ubiquitinated protein was described previously [44,60]. Briefly, cells were lysed with radioimmunoprecipitation assay (RIPA) lysis buffer [50 mM tris-HCl (pH 7.4), 150 mM NaCl, 1 mM EDTA, 0.25% deoxycholic acid, and 1% NP-40] containing 2% SDS, 1 mM DTT, 10 mM N-Ethylmaleimide (NEM), and protease and phosphatase inhibitors cocktail (Roche), and then boiled at 95°C for 10 min, followed by brief sonication until a clear solution was obtained. Cell lysates were then centrifuged at maximum speed for 10 min, the supernatants were diluted 10 times with additional RIPA lysis buffer to reduce the SDS concentration to 0.2%, and then immunoprecipitated using anti-HA M2 affinity Gel (Sigma, #E6779). After 4 h incubation at 4˚C, the beads were washed for three times with WCL and twice with PBS, and then boiled with the 2 x SDS loading buffer for 10 min. The immunoprecipitants were applied to standard immunoblotting analyses with specific antibodies.

### Virus infection

EBV-positive Akata cell line was kindly provided by Dr. Musheng Zeng from Sun Yat-sen University and HCMV was kindly provided by Dr. Minhua Luo from Wuhan Institute of Virology. KSHV-positive iSLK.r219, iSLK-BAC16, SLK-iBAC, and BCBL-1 cells were described previously [44,57,61–65]. Akata cells were harvested by centrifugation, resuspended in fresh complete RPMI 1640 medium and adjusted to a density of 3 × 10^6^ cells/mL. The cells were then treated with 10 μg/mL of goat anti-human IgG antibody. The cells were harvested at 48 h post-induction, and the culture supernatants were collected at 72 h post-induction for further analysis. THP-1 cells were first harvested by centrifugation and resuspended in viral-enriched culture medium at a multiplicity of infection (MOI) of 2. THP-1 cells were then seeded into six well plates and spin-inoculated at 800 × g for 45 min. The latently infected THP-1 cells were treated with 100 nM TPA and plated on tissue culture-treated plates to promote monocyte-to-macrophage differentiation. The differentiated adherent cells were incubated at 37°C for 96 h post-induction and the culture supernatant was harvested for further analysis.

### RNA purification and RT-qPCR

Cells were seeded in 12-well plate and over 10^6^ cells were collected for RNA extraction. Total RNA was extracted from cells with TRIzol reagent (Sigma) according to the manufacturer’s protocol. Briefly, 1 μg of total RNA was reverse transcribed by HiScript II Q RT SuperMix for qPCR (+gDNA wiper) (Vazyme Biotech, #R223-01) and the cDNA was quantified by SYBR green (Vazyme, #Q312-02) based qPCR using gene specific primers. The relative level of gene expression was calculated by the fold change (2^-ΔΔCt^) between the experimental samples and the control, while GAPDH was used for normalization. The RT-qPCR graphs represent the average of at least three independent experiments. The sequences of the primers used in RT-qPCR have been described previously [44,45,66].

### Quantification of intracellular and extracellular virion genomic DNA

SLK-iBAC and its derived cells were induced with doxycycline (2 μg/mL) and sodium butyrate (1 mM) for KSHV reactivation, BCBL-1 cells were treated with TPA (20 ng/mL) for KSHV lytic replication. Total intracellular DNA was purified with a cell DNA isolation Kit (Vazyme Biotech, #DC102–01) according to the manufacturer’s instructions. The viral DNA was measured by qPCR using primers for ORF11 (Fw-GGCACCATACAGCTTCTACGA and Rev-CGTTTACTACTGCACACTGCA) and normalized to β-actin (Fw-CGGGAAATCGTGCGTGACATT; Rev-CAGGAAGGAAGGCTGGAAGAGTG). The culture supernatants were centrifuged and passed through a 0.45-μm filter to remove cellular debris. The clarified supernatants were treated with DNase I to degrade unencapsidated host and viral DNA. Extracellular supernatant viral DNA was purified with a viral DNA isolation Kit (Vazyme Biotech, #RC311). The genomic DNA in virions was measured by qPCR with specific primers. Viral DNA copy numbers were calculated with external standards of known concentrations of serially diluted BAC16 DNA (KSHV) or recombinant plasmid construct containing the viral target gene sequence (EBV and HCMV) ranging from 1 to 10^7^ genome copies per reaction. The KSHV genomic DNA in virions was measured with primers ORF11. The EBV genome copy number was quantified by qPCR targeting the viral BALF5 gene (primers: Fw-ACCTCAGCGTGGAGATTGTG and Rev-CCAGAGAGGCTGGGTTGATG). The HCMV genome copy number was quantified by qPCR targeting the viral UL83 gene (primers: Fw-GATGCGATACTGGCTGGTGAAG and Rev-GAGGTACAAGCCATACGCGAGA).

### Quantification and statistical analysis

All data were expressed as Mean ± s.d., unless otherwise noted. For parametric analysis, the F test was used to determine the equality of variances between the groups compared; statistical significance across two groups was tested by two-tailed unpaired Student’s t-test; one-way analysis of variance (ANOVA) followed by Bonferroni’s *post hoc* test were used to determine statistically significant differences between multiple groups. *P*-values of less than 0.05 were considered significant.

## Supporting information

S1 Raw gel Raw data for western blot images. (PDF)
