# Supplementary material for: The TRAF6-Akt axis is required for efficient lytic replication of KSHV
Source: PLoS Pathog. 2026 Sep 1;22(9):e1014557. doi: 10.1371/journal.ppat.1014557 (PMC13561422; doi:10.1371/journal.ppat.1014557)

Figure 1

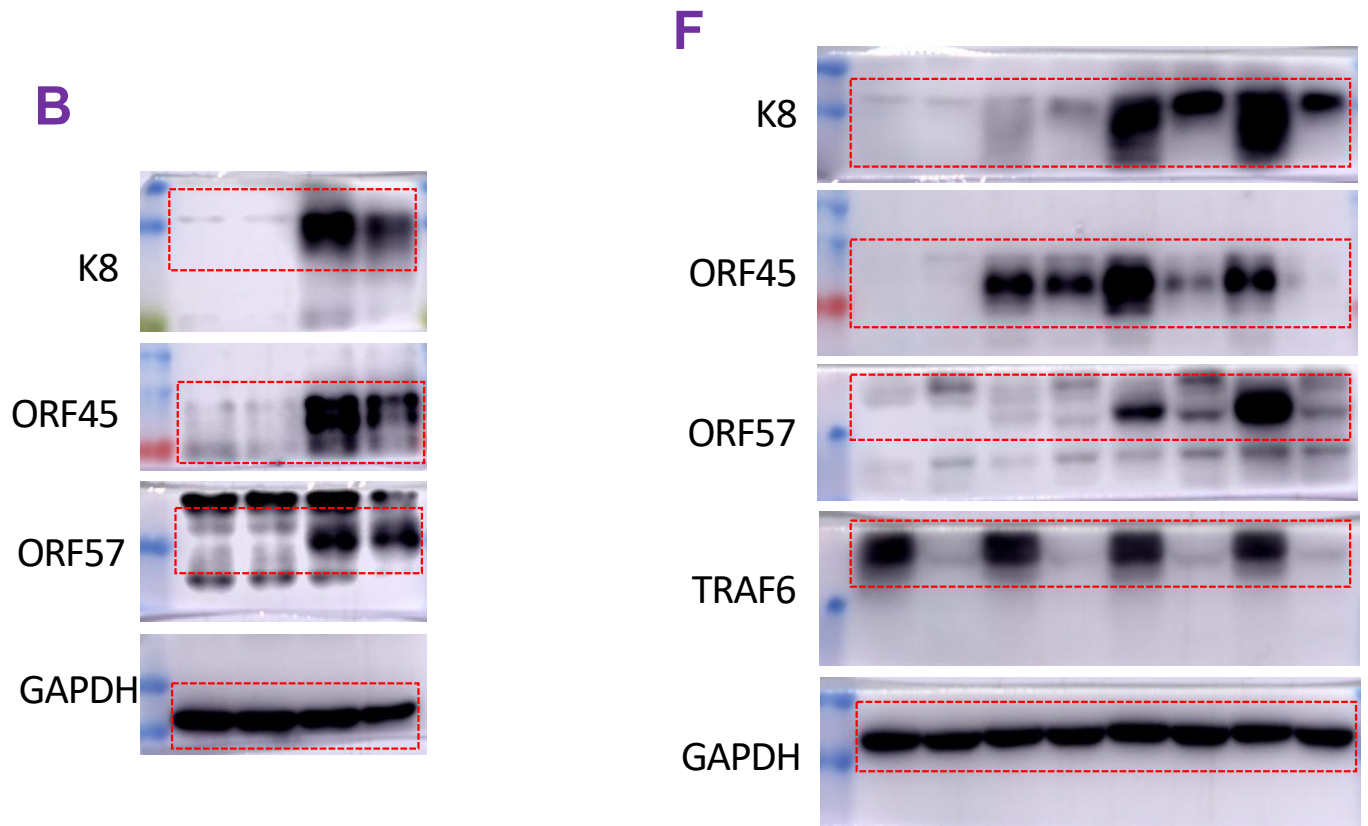

Figure 2

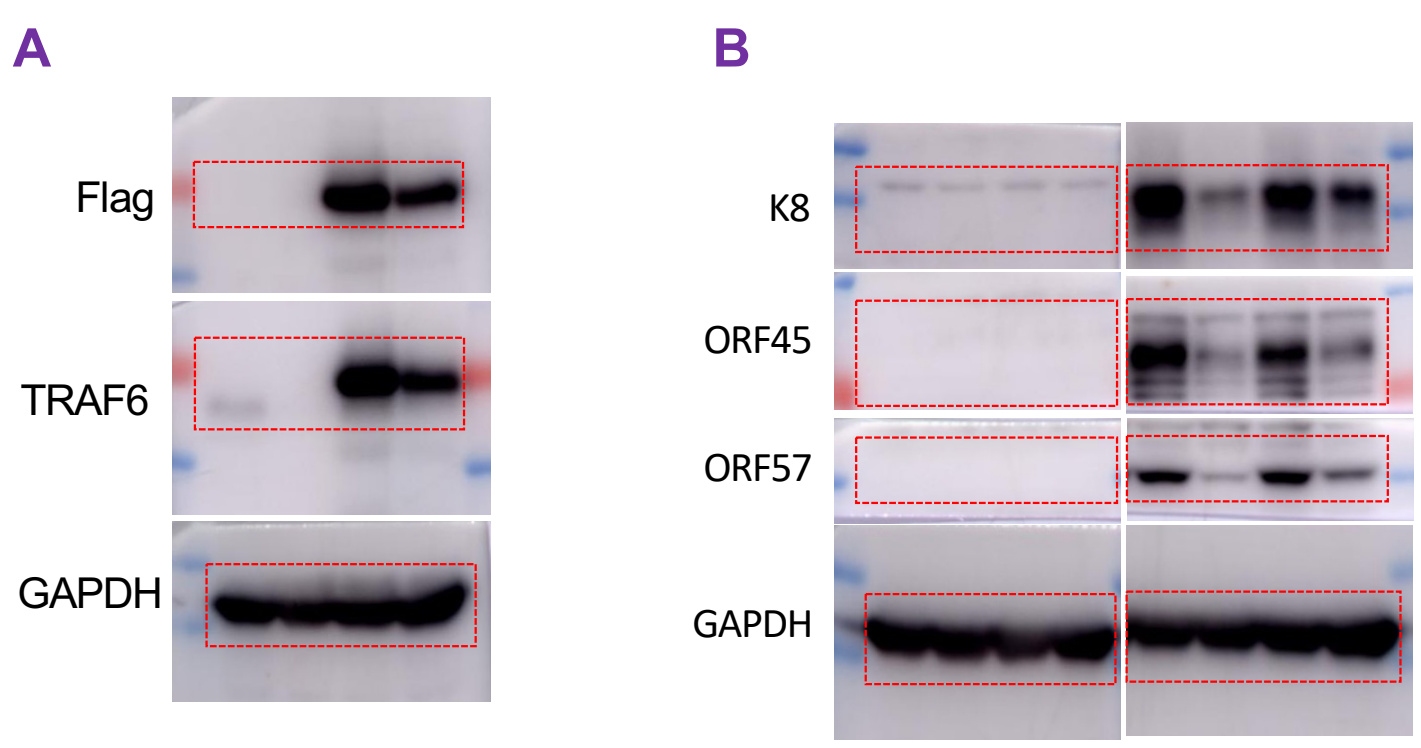

Figure 3

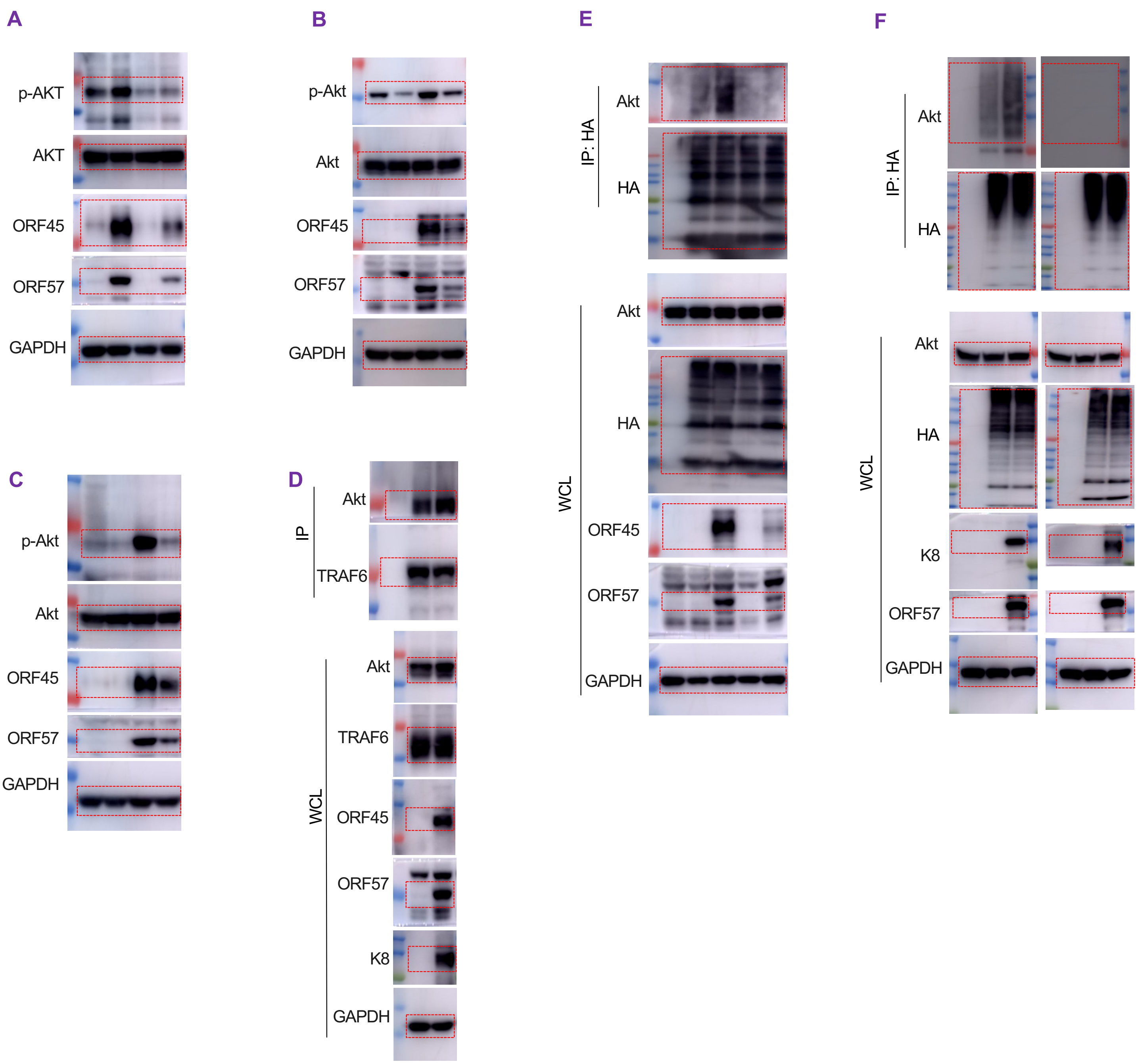
A
p-AKT
AKT
ORF45
ORF57
GAPDH
B
p-Akt
Akt
ORF45
ORF57
GAPDH
C
p-Akt
Akt
ORF45
ORF57
GAPDH
D
IP
Akt
TRAF6
WCL
Akt
TRAF6
ORF45
ORF57
K8
GAPDH
E
IP: HA
Akt
HA
WCL
Akt
HA
ORF45
ORF57
GAPDH
F
IP: HA
Akt
HA
WCL
Akt
HA
K8
ORF57
GAPDH

Figure 4

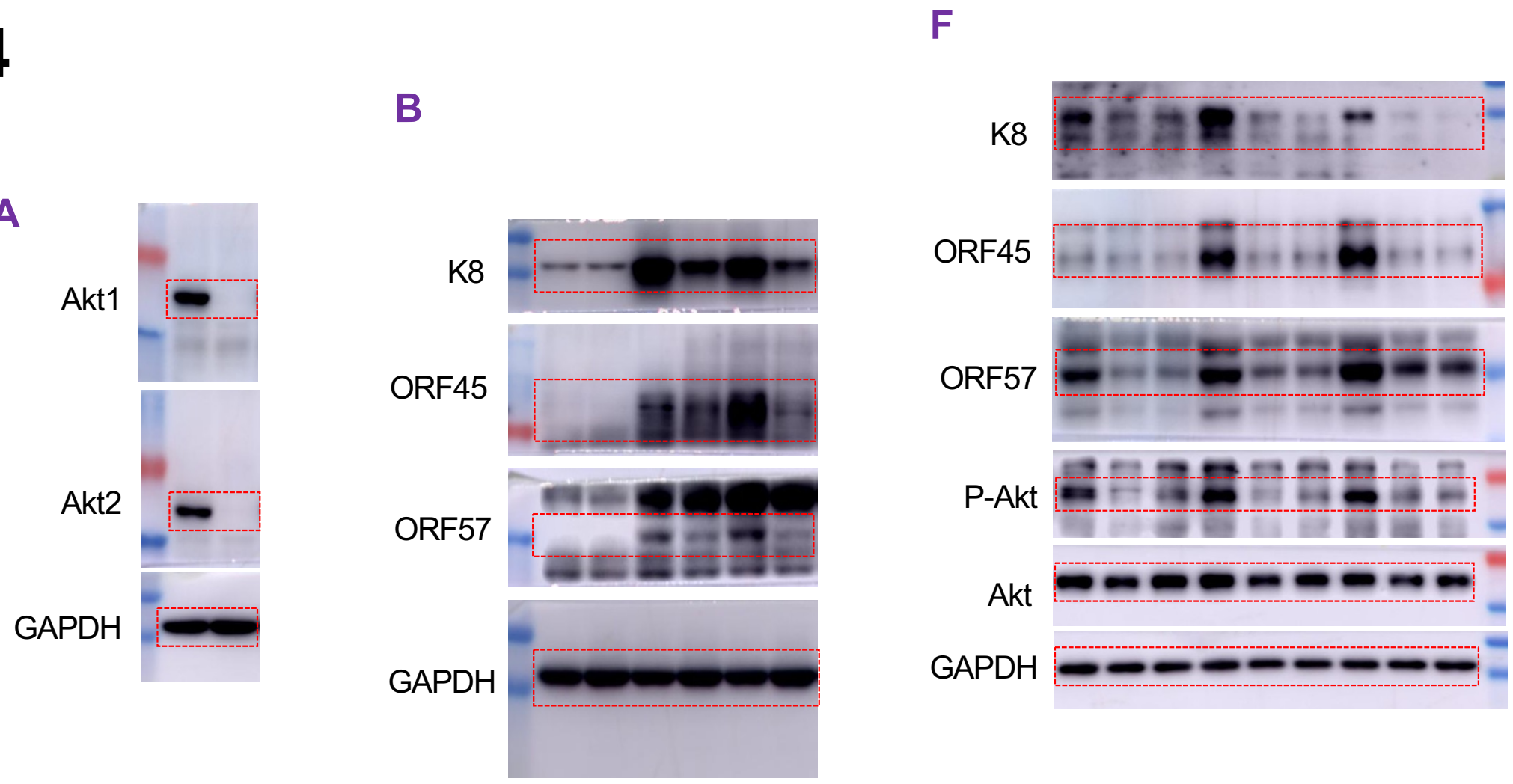

Figure 5

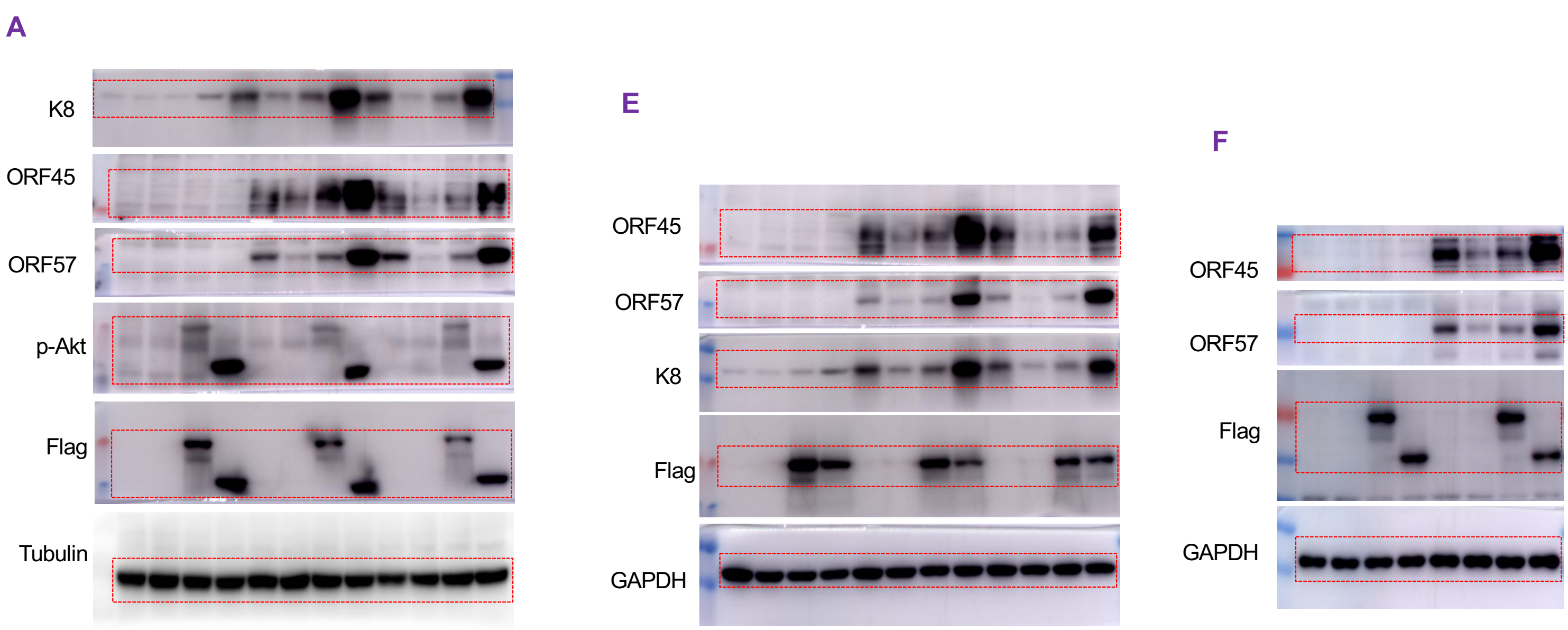

Supplement: Supplementary file 1 [file ppat.1014557.s001.pdf]
